# Neuronal ceroid lipofuscinoses type 7 (CLN7): a case series reporting cross sectional and retrospective clinical data to evaluate validity of standardized tools to assess disease progression, quality of life, and adaptive skills

**DOI:** 10.1186/s13023-024-03448-8

**Published:** 2024-12-19

**Authors:** Saima Kayani, Veronica BordesEdgar, Andrea Lowden, Emily R. Nettesheim, Hamza Dahshi, Souad Messahel, Berge A. Minassian, Benjamin M. Greenberg

**Affiliations:** 1 Department of Pediatrics, University of Texas Southwestern Medical Center, 5323 Harry Hines Blvd, Dallas, TX 75390 USA; 2 Children’s Health Dallas, Dallas, TX USA; 3 Department of Psychiatry, University of Texas Southwestern Medical Center, Dallas, TX USA; 4 Department of Neurology, University of Texas Southwestern Medical Center, Dallas, TX USA; 5 Perot Neuroscience Translational Research Center, Peter O’Donnell Brain Institute, Dallas, TX USA; 6 Department of Ophthalmology, University of Texas Southwestern Medical Center, Dallas, TX USA

**Keywords:** MFSD8, Neuronal ceroid lipofuscinosis, Batten disease, Rare brain disease

## Abstract

**Background:**

This study evaluated the clinical characteristics of neuronal ceroid lipofuscinosis type 7 or CLN7 disease spectrum to characterize the clinical, electrophysiologic and neuroimaging phenotypes.

**Methods:**

We performed a single-center cross sectional data collection along with retrospective medical chart review in patients with a genetic diagnosis of CLN7. This study received ethical approval by the University of Texas Southwestern Medical Center Institutional Review Board. A total of 8 patients were included between the ages of 4 to 6 years. All patients had a genetic diagnosis of CLN7 with homozygous or compound heterozygous mutations in the *MFSD8* gene. The information collected includes patient demographics, developmental history, neurological events including seizures and neurodevelopmental regression along with further evaluation of brain magnetic resonance imaging and electrophysiological findings. The clinical phenotype is described through cross sectional and retrospective data collection and standardized tools assessing quality of life and functional skills.

**Results:**

Our findings in this cohort of CLN7 patients indicated that development is initially normal with onset of clinical symptoms as early as two years of age. Language problems were noted prior to or at the onset of seizures in all cases. Gait problems were noted prior to seizure onset in 3 of 8 patients, and at or within 6 months after the onset of seizures in 5 of 8 patients. All patients followed a progressive course of language, motor, and neurocognitive deterioration. Congruent with the medical history, our patients had significantly low scores on adaptive abilities. Natural history data such as this can be used to support future clinical trial designs.

**Conclusions:**

This study provides a comprehensive description of CLN7 disease, highlighting clinical data alongside standardized neuropsychological assessments, neuroimaging, and electrophysiologic data. It emphasizes the value of importance of standardized tools for understanding disease phenotype and their potential use as endpoints in future clinical trials. The findings established can provide a baseline for developing future prospective natural history studies and potential therapeutic clinical trials.

**Supplementary Information:**

The online version contains supplementary material available at 10.1186/s13023-024-03448-8.

## Introduction

Recent advances in gene therapy have enabled development of potentially disease-modifying therapies for monogenic neurodegenerative diseases like neuronal ceroid lipofuscinosis type 7 (CLN7).

CLN7 is an autosomal recessive disorder which is caused by homozygous or bi-allelic heterozygous variants in the CLN7/MFSD8 gene. This gene is typically inherited from healthy, carrier parents each contributing a defective copy. The *CLN7/MFSD8* gene encodes a 518-amino acid polytopic lysosomal transmembrane protein with 12 membrane-spanning domains [1, 2]. After the initial identification of the *MFSD8* gene in 2007, a total of 67 different *MFSD8* mutations have been reported in populations throughout the world (Table 1 in supplementary materials). The types of mutations include missense, splice site, nonsense, frame shift, sequence deletion or insertion [3]. The clinical spectrum can vary from a mild, late onset with non-syndromic visual deficits [4] to a severe, early-onset version that can manifest with progressive deterioration in intellectual and motor capabilities, seizures, muscle spasms and visual deficits culminating in premature death. [5]

## Materials and methods

### Study design and population

Ethical approval was obtained from the University of Texas Southwestern Medical Center Institutional Review Board prior to commencing this research study (STU 2020-0916, STU 2020-0640). An informed consent waiver was obtained for all participants. We performed a single-center cross sectional data collection and a retrospective medical chart review that included 8 patients with genetic diagnosis of CLN7. Data was collected during a natural history study of CLN7 for four patients over the course of 1 year. Baseline assessments and retrospective clinical data is reported alongwith neuropscyhological assessments. The remaining four patients are currently seen in our center as part of a gene therapy clinical trial. For the patients enrolled in the clinical trial, baseline clinical assessments are reported along with retrospective data that was collected before the initial screening visit except for neuropsychological assessments where an additional datapoint was added on two measures.

Patients were eligible for the study if they were under the age of 18 years and had a confirmed genetic diagnosis of CLN7, defined as two pathogenic variants in the *MFSD8* gene. In addition, patients enrolled in clinical trial had to meet the inclusion criteria that specified the enrollment of patients with symptomatic disease but not advanced disease. To be eligible, patients either were to have an age equivalent expressive language score of 2 year and 0 month on Mullen or Vineland language sub domain or patients had to obtain a score of 2–3 on Gross Motor Function Measure sub domain E (Walking, Running and Jumping) item 67. The complete inclusion and exclusion criteria is listed in clinicaltrials.gov (NCT04737460). Collected data included longitudinal sociodemographic characteristics, brain MRI (Magnetic Resonance Imaging) findings, clinical presentations, laboratory results, operative reports if available, and medications. Motor and cognitive development was also reviewed, and data was collected on motor functions and intellectual ability, by standardized neuropsychological assessments. CLN7 mutation type was obtained from medical records and reviewed by study investigator and was additionally confirmed by repeat molecular tesing for patients enrolled in clinical trial. Additional data including age of symptom onset, age of diagnosis, regression (motor, language, or cognitive), presenting neurologic signs and symptoms, and developmental milestones were collected based upon parent report and corroborated through previous medical records when available. Electrophysiologic data were collected per clinical standards and EEG data were analyzed by a board-certified epileptologist.

Participants were assessed by a board-certified neuropsychologist who administered standardized measures with both the child and their caregivers. Individual assessments with the children were done in their native language either through the bicultural/bilingual neuropsychologist (certified in Spanish) or a professional interpreter when English or Spanish was not the native language. Professional interpreters received training instructions in how to assist in the evaluation. The following measures were administered in this order: Mullen Scales of Early Learning (Mullen) [6], Vineland Adaptive Behavior Scales, 3rd Edition Comprehensive Interview (Vineland-3) [7], Infant Toddler Quality of Life Questionnaire (ITQOL) [8] for children under 5 years and the Quality of Life Inventory-Disability (QI-Disability) [9, 10] for children 5 years of age and older. The natural history patients (participants 1–4) only received the Mullen and Vineland-3. Tests were selected to accurately assess each patient based on the child’s individual abilities and stamina even when used outside of the age range (i.e., Mullen).

The Mullen is a test of early cognitive ability and motor development. It is made up of five scales: Gross Motor, Fine Motor, Visual Reception (visual problem-solving), Receptive Language and Expressive Language. The latter four make up the Early Learning Composite [6]. The raw scores were converted to age equivalents and subsequently to developmental quotients (age equivalent/chronological age × 100) as opposed to norm-referenced scores. While this test has limitations in the age of data and test items, this test was selected over other measures due to its brevity and ease in administration with more impaired participants. The normative data, although dated, extends to 68 months which is further than other infant measures. Given the decline in participants, raw data and age equivalents are utilized to capture the decline or progress in development of participants. Given the low functioning of participants, standard scores are rarely used, therefore, Flynn effect and other concerns with using dated norms do not apply to measured change over time. The Mullen also allows the researchers to separate out expressive and receptive language abilities as well as motor functioning which may have differential impact based on the disease process. Further, vision loss in participants will contribute to performance across any standardized measure making the separation of language and motor functioning from problem-solving even more necessary.

Adaptive behavior can be defined as the performance of daily activities required for personal and social sufficiency. Adaptive behavior is impacted in virtually all disorders that cause cognitive/developmental regression. The Vineland-3 [7] was utilized to assess adaptive behavior. This test was administered in a structured interview format with the participant’s caregivers to reduce the impact of parental reporting bias on scores. Scores are norm-referenced standard scores for the domains (SS mean = 100, SD = 15) and v-scale scores for sub-domains (mean = 15, SD = 3). Further, developmental quotients were also calculated for language and motor scales.

Infant Toddler Quality of Life Questionnaire (ITQOfL) [8]- Short form is a 47-item measure to assess physical and psychosocial functioning of children ages 2 months to 5 years. It contains 12 scales including physical functioning, growth and development, bodily pain, temperament and moods, general behavior, getting along, general health perceptions, parental impact: emotional, parental impact: time, family activities, family cohesion, and change in health. Scores transformed to scale of 0 to 100 with higher scores indicating better quality of life.

Quality of Life Inventory-Disability is a measure to capture the health and well-being of children ages 5–18 with intellectual disability (QI-Disability) [9, 10]. This measure was particularly developed to assess children with a wide range of disabilities and has been validated on children with Rett Syndrome, Down Syndrome, and CDKL5 deficiency disorder. Both Rett Syndrome and CDKL5 deficiency disorder are most like our study population. The validation on children with CDLK5 deficiency disorder spanned children as young as 3 years of age. This 32-item measure assesses a child’s social interactions, positive emotions, physical health, negative emotions, leisure skills, and independence. Scores are a 5-point Likert scale and transformed to scale of 0–100 with higher scores indicating better quality of life.

Given this is an ultra-rare disease, we wanted to be inclusive of participants regardless of linguistic and cultural differences. This is reflected in the diverse geographic and cultural representation of participants. Cultural and linguistic considerations were made across measures including the use of interpreters. Performance-based measures (i.e., Mullen) included nonverbal or very brief instructions given the task items that were being administered. Parents also remained in the room as is appropriate with the Mullen and were able to validate the child’s performance as consistent with their current functioning. The adaptive measure (i.e., Vineland-3) is also scored based on clinician judgment from the interview questions asked. This reduces the reliance on the interpreter needing to interpret verbatim and allows for tailoring questions to the parents in a way they will understand. While the same interpreter was not always available for follow-up evaluations, the same examiner was used which allowed for consistency in the clinical judgment. Possible bias was reduced on the part of the examiner as scores from previous evaluations are not available to the examiner after they are submitted to the study team and medical records are not reviewed. See our other work for more detailed information in the cultural considerations [11].

A 21-channel video-EEG recording was obtained and analyzed in each patient. Electrodes were placed using the 10–20 nomenclature system. Photic stimulation was performed from 1 to 20Hz with 10 s trials and testing with the eyes opened and closed. The recording was of at least 60 min in duration in all 8 patients. For all the patients, EEG data were interpreted by a single board certified epileptologist.

MRI brain study was done on a 3 T Siemens Skyra scanner with software version V11E.

The pulse sequences include localizer images and the following:

1. MPRAGE (3D sagittal T1 with TR/TE/TI = 2500/3.11/1240 ms; flip angle = 8 degree, slice thickness = 1 mm, in plane acquisition resolution 0.8 mm and reconstructed into 0.4 mm pixel size, acquisition time 5:14 min:sec);
2. Diffusion tensor and kurtosis imaging (b = 1000, 2000s/mm^2^; voxel size 3 × 2.7 × 2.7 mm^3^, 30 directions for each shell, acquisition time 12:14 min:sec);
3. 3D T2 FLAIR (TR/TE/TI = 5000/394/1800 ms, slice thickness = 1 mm, in plane acquisition resolution 1 mm and reconstructed into 0.5 mm pixel size, acquisition time 6:07 min:sec);

MRI data were interpreted by pediatric neuro-radiologist on call for the day.

### Data collection

Electronic medical records from UTSW/Children’s Health Dallas with a genetically confirmed diagnosis of CLN7 were reviewed. Deidentified data were abstracted into a password protected spreadsheet for analysis.

## Results

In the cohort under study, a total of eight patients were enrolled, with ages spanning from 4 to 6 years. The studies group participants consisted of 5 females and 3 males. All the patients included in this study were from distinct families and were unrelated to each other. The collected dataset is described below and encompasses the demographic information, genotypic profile, key clinical, neuroimaging and electrophysiologic characteristics. Among the clinical phenotypic spectrum, our group focused on studying the onset and progression of neurologic symptomatology and comprehensive neuropsychological evaluation.

### Demographics/genotype

All 8 patients were clinically diagnosed with CLN7 deficiency, and the diagnosis was molecularly confirmed with homozygous or bi-allelic heterozygous pathogenic mutations in *MFSD8* gene. Analysis of the *MFSD8* gene mutations in our patient population revealed 12 distinct pathogenic variants, out of which 6 variants (Table 1) are novel to this study and have not been described in literature before.

**Table 1:** Demographics- participants gender, country of origin and genotype

Subject ID	Gender	Country/language	Affected gene	Mutation information	Zygosity	Predicted DNA variation
1	Female	India-USA/English	MFSD8	c.1351-2A > G^†^	Homozygous	Splice acceptor
2	Male	USA/English	MFSD8	c.1036delG (p. Val346LeufsTer68)^†^; c.440-2A > T^†^	Compound Heterozygous	Frameshift Splice acceptor
3	Female	Sweden/Swedish	MFSD8	c.1437G > A, (p.Trp479Ter)^†^; c.1444C > T (p.Arg482Ter)	Compound Heterozygous	Stop gain Stop gain
4	Male	Hungary/Hungarian	MFSD8	c.881C > A p.(Thr294Lys); c.754 + 2 T > A	Compound Heterozygous	Missense Splice donor
5	Female	Paraguay/Spanish	MFSD8	c.103C > T (p.Arg35Ter)	Homozygous	Stop gain
6	Male	Romania/Romanian	MFSD8	c.1373C > A (p.Thr458Lys);	Homozygous	Missense
7	Female	Saudi Arabia/Arabic	MFSD8	c.198 + 2 T > C^†^	Homozygous	GT Splice donor
8	Female	USA/English	MFSD8	c.1444C > T (p. Arg482Ter); c.1206del (p.Ile403LeufsTer11)^†^	Compound Heterozygous	Stop gain Frameshift

^†^Denotes *MFSD8* gene mutations not previously reported in the literature

The genetic diagnosis was made through epilepsy gene panels, whole exome sequencing and *MFSD8* sequence analysis. Descriptive analysis was used to analyze the data which revealed heterogeneity of geographic, cultural, and linguistic and demographics backgrounds as detailed in Table 1.

### Clinical characteristics

#### Birth history, early development, and age of onset of symptoms

All patients (n = 8) were reported to have an uncomplicated birth and an unremarkable early post-natal period. Early development was normal until 2 years of age.

The earliest symptom onset was at two years of age. In all the patients (n = 8) the onset of symptoms was noted before 4 years of age (Table 2) and the age of diagnosis was between 4 and 5 years. In our study cohort, median age of onset of symptoms is 3.4 years and median age of diagnosis is 4.8 years.

**Table 2:** Developmental regression

Subject ID	Age at visit (yy.mm)	Age at onset of first symptom (yy.mm)	Age at diagnosis (yy.mm)	Initial symptom	Seizure onset (yy.mm)	Onset of gait problems (yy.mm)	Onset of language difficulties (yy.mm)	Onset of cognitive issues (yy.mm)	Onset of vision changes (yy.mm)
1	6.9	3.4	4.4	Gait Problems	4	3.5	4	3.5	5
2	6.11	4	4.75	Vision change	4.4	4.5	2.5	Unsure	4
3	5.5	2.4	5	Gait problems	4.8	2.5	2.5	3.5	2.5
4	5.6	3	5	DD	3.5	Unsure	2	4.5	No vision problems
5	5	2	4.4	Language issue	3.5	3.5	2	3.5	Un-determined
6	4.7	3.8	4	Shaking/fall	3.8	Intermittent with myoclonus at onset of symptoms	3.5	Unsure	Un-determined
7	5.11	3.75	5.25	Shaking/falls	4	4.5	2	Unsure	Un-determined
8	4.1	3	4	Paucity of language and motor development	3.5	2.5	2.5	2–3	Un-determined

In the study cohort, the initial presenting symptoms prompting the families to seek medical attention varied among the patients. Per parent report, 2 out of 8 patients presented with gait disturbance as the initial symptom, while another 2 exhibited signs of tremors and recurrent falls. Additionally, one patient presented with vision changes, and difficulties in language were reported in 2 patients, either in terms of articulation or fluency and 1 patient was brought to medical attention because family was concerned about developmental delays, which was described as an overall concern about the lack of progress in neurologic development specifically in language and fine motor domains.

It is noteworthy, that the first clinical symptoms, prompting the family to seek medical attention is parent reported and is described here as it was noted in medical records. It is important to mention that detailed clinical and neuropsychological assessments were not conducted at the time of onset to provide detailed characterization of specific nature and extent of language difficulties or developmental delays.

Another noteworthy observation emerged during retrospective analysis, that the majority of patients, 6 out of 8 to be specific, had a paucity in language development, occurring at or right after 2 years of age. This plateau in language development, however, did not attain a level of significance which would either prompt families to seek medical attention or alert the primary care physician to pursue further diagnostic testing.

#### Neurological symptoms

##### Developmental regression

Gait Problems

**Fig. 1:**
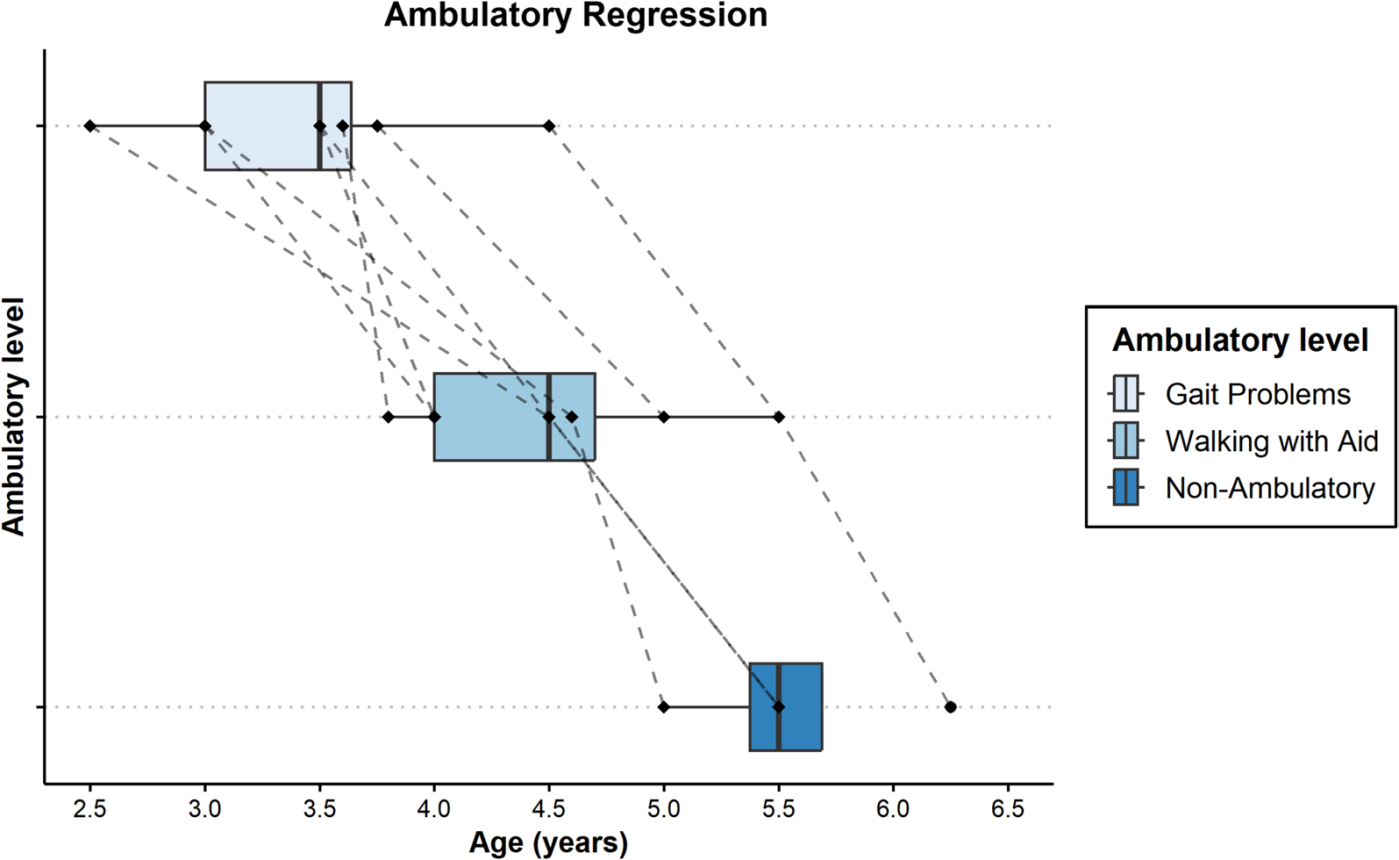
Box plots of the age of onset for stages of ambulatory regression

Gait disturbance was reported by families as early as 2 and a half years of age and as late as 4 and half years of age. Gait progressively deteriorated requiring an aid to walk within 1–2 years from onset of gait difficulty (Table 2). Once walking difficulties were noticed, the earlier level of function was never obtained. All the patients who were seen at advanced disease stage were non-ambulatory within 1 year of age when they needed help with walking (Fig. 1).

Language difficulties

Language difficulties or delays were noted as early as 2 years of age in 3/8 patients, 2.5 years in 3/8 patients, 3.5 years in 1/8 patients and by 4 years in 1/8 patients. All the patients at the time of visit had moderate to severe language issues.

##### Seizures

In this patient cohort the onset of seizures ranged from 3 years and 5 months to 4 years and 10 months. Various seizure types were reported including myoclonic, atonic, and bilateral tonic–clonic seizures. In addition to seizures, myoclonic jerks were reported in all but one study participant. One unprovoked seizure was captured during EEG, identified by the caregiver, and characterized clinically by a brief head drop (atonic seizure).

In this study cohort, the majority (6 out of 8) of patients required more than one anti-seizure medication to achieve adequate seizure control. The most common anti-seizure medication used in this study cohort was Valproic acid, and 6 out of 8 patients were taking Valproic acid. In one patient, Valproic acid was stopped later in the disease course due to ineffectiveness and replaced with an alternative medicine. Clobazam and Levetiracetam were the second most common medications used and 5 out of 8 patients were taking these anti-seizure medications. Lamotrigine was also used for seizure control in 2 out of 8 patients in this cohort. Cannabidiol was used in 2 out of 8 patients. One study patient was on Rufinamide and Clonazepam. There was no clear benefit of one anti-seizure medication over the other, however, this data are not sufficient to establish such a correlation. One patient had excessive sedation on combination of Clobazam and Cannabidiol (FDA approved prescribed formulation used), however there were no other harmful effects or side effects reported for any other anti-seizure medication at the time of the study. It should be noted that one of the limitations of the retrospective study is paucity of data and a complete profile of longitudinal use of anti-seizure medications along with the side effects and interactions of various medications is not available to report in some cases.

Figure 1 shows box plots of the age of onset for stages of ambulatory regression. Stages range from gait problems (light blue) down to non-ambulatory status (dark blue). Ages range from roughly 3 years of age to around 6 years of age. Each line corresponds to one of the 8 individual patients.

### Neuro-imaging findings

Abnormal MRI results were observed in all (8/8) cases reviewed. All patients had generalized findings with diffuse cortical and cerebellar atrophy with white matter involvement (see Table 3). Diffuse white matter involvement with T2 hyperintensities were noted in 7/8 cases and periventricular gliosis was reported in 2/8 cases. Thalami were involved in all (8/8) cases with a variable degree of volume loss and gliosis. The neuroimaging data was compared to the previous scans for cases where the previous reports or images were avaialble.

**Table 3:** Neuro-imaging findings

Subject ID	Age (years. months)	MRI brain
1	6.9	Significant progressive cerebral and cerebellar atrophy Faint regions of T2 signal hyperintensity in the periventricular white matter Bilateral thalamic atrophy
2	6.11	Generalized parenchymal volume loss and periventricular gliosis White matter pallor on T2 Flair images with increased signal intensity in periventricular white matter and posterior limbs of internal capsule Thalamic atrophy and gliosis
3	5.5	Diffuse supratentorial and infratentorial atrophy White matter appropriately myelinated Mild thalamic volume loss with gliosis
4	5.6	Generalized parenchymal volume loss Periventricular gliosis Paucity of white matter Increased T2 signal abnormalities along periventricular white matter Thalamic volume loss
5	5	Mild to moderate generalized cerebral and cerebellar atrophy associated Abnormal myelin pallor involving the deep cerebral white matter and corticospinal tracts Marked thalamic atrophy
6	4.7	Generalized mild cerebral and moderate cerebellar atrophy. Confluent myelin pallor involving the deep cerebral white matter and extending along the corticospinal tracts Moderate thalamic atrophy
7	5.11	Mild to moderate generalized cerebral and cerebellar atrophy. Moderate ventriculomegaly White matter paucity Mild myelin pallor in the periventricular white matter
8	4.1	Mild to moderate generalized cerebral atrophy with nonobstructive ventricular enlargement Symmetric myelin pallor involving the deep cerebral white matter with moderate bilateral thalamic atrophy

#### Electrophysiological findings

**Fig. 2:**
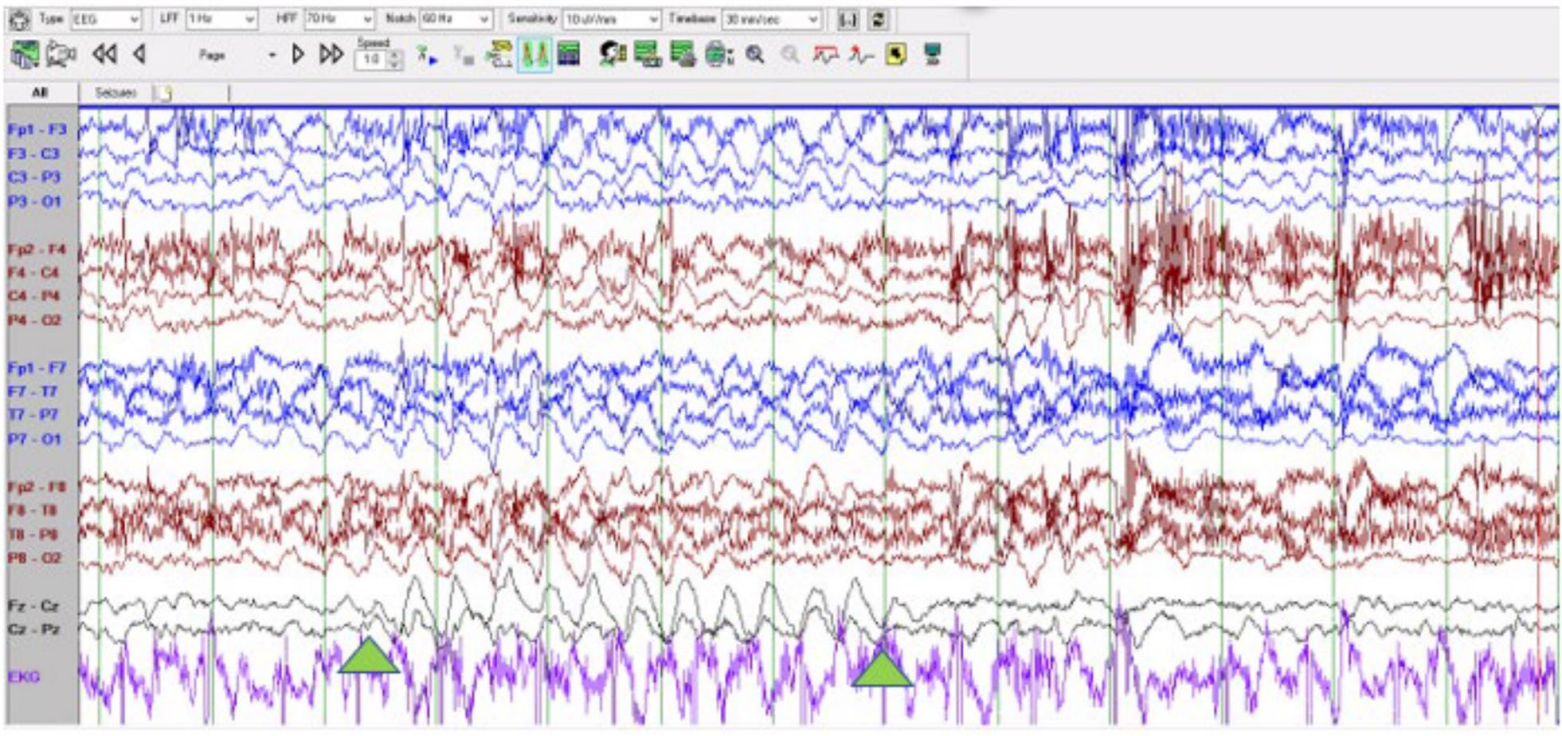
Interictal background showing generalized rhythmic delta activity

Electroencephalographic interictal background activity for all eight patients was abnormal. Interictal background activity showed continuous generalized delta and theta slowing in 37.5% and 62.5% of patients, respectively. In 5 out of 8 patients generalized rhythmic delta activity was seen which often was maximal in the bioccipital or bifrontal regions (Fig. 2). Independent multifocal spikes (100% of patients) along with generalized (75% of patients) with posterior maximum epileptiform discharges were seen. During photic stimulation no electroretinogram artifact, photomyoclonus or photoparoxysmal responses were seen in 6 of 8 patients. In one patient photic stimulation triggered a focal motor (myoclonic) seizure with concomitant spike and wave activity seen in the bioccipital regions. One unprovoked seizure was captured, identified by the caregiver, and characterized clinically by a brief head drop (atonic seizure). Ictal EEG showed a generalized spike and wave correlate. One event of non-epileptic staring was seen with no electrographic correlation.

Figure 2 This figure illustrates the EEG recording of a patient during the interictal period. The data depict a consistent pattern of generalized rhythmic delta activity, characterized by a regular, rhythmic waveform. The presence of rhythmic delta waves in the interictal phase is indicative of potential underlying neurological conditions

#### Neuropsychological assessment results

**Fig. 3:**
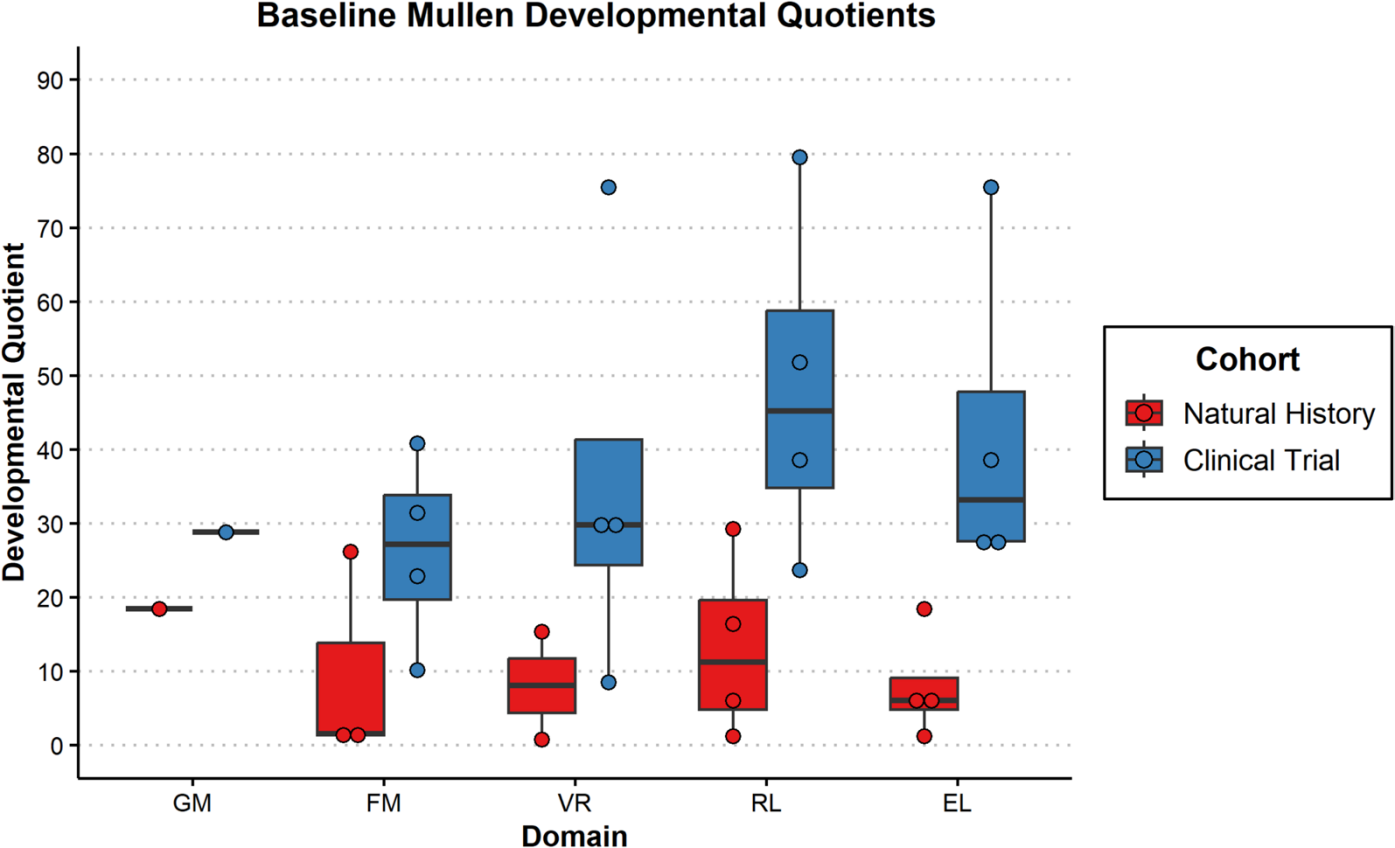
Boxplots of developmental quotients from the mullen for subjects 1–8

Assessment of developmental/cognitive abilities was limited for this population given advanced disease progression. Of the 8 children seen, 6 had complete Mullen administrations (participants 3–8). The other two were both evaluated on receptive and expressive language and only one (participant 1) on fine motor abilities. Except for participant 8, all others obtained a standard score at the floor (i.e., lowest score available) of the measure (Standard Score = 49) and participant 8 performed in the exceptionally low range (Early Learning Composite Standard Score = 63). Additionally, participants 1, 2, and 7 were outside of the normative age-range. As such, Developmental Quotients are thought to best capture the participants’ functioning and are presented in Fig. 3. Participants 5 and 6 underwent 6-month follow-up evaluations with the Mullen demonstrating regression in all domains for both participants (Fig. 1 in supplementary Materials. The slope of the regression was most significant in the area of receptive language (− 0.13 participant 5 and − 0.45 participant 6) compared to fine motor (− 0.02 participant 5 and − 0.18 participant 6), visual reception (− 0.08 participant 5 and − 0.15 participant 6), and expressive language (− 0.16 participant 5 and − 0.21 participant 6) slopes.

Figure 3 Boxplots of Developmental Quotients from the Mullen for subjects 1–8. The scores represented are the Gross Motor (GM), Fine Motor (FM), Visual Reception (VR), Receptive Language (RL), and Expressive Language (EL) subtests. Subjects from the natural history study (NH) are colored red, and subjects from the gene therapy clinical trials (Clinical) are colored blue

**Fig. 4:**
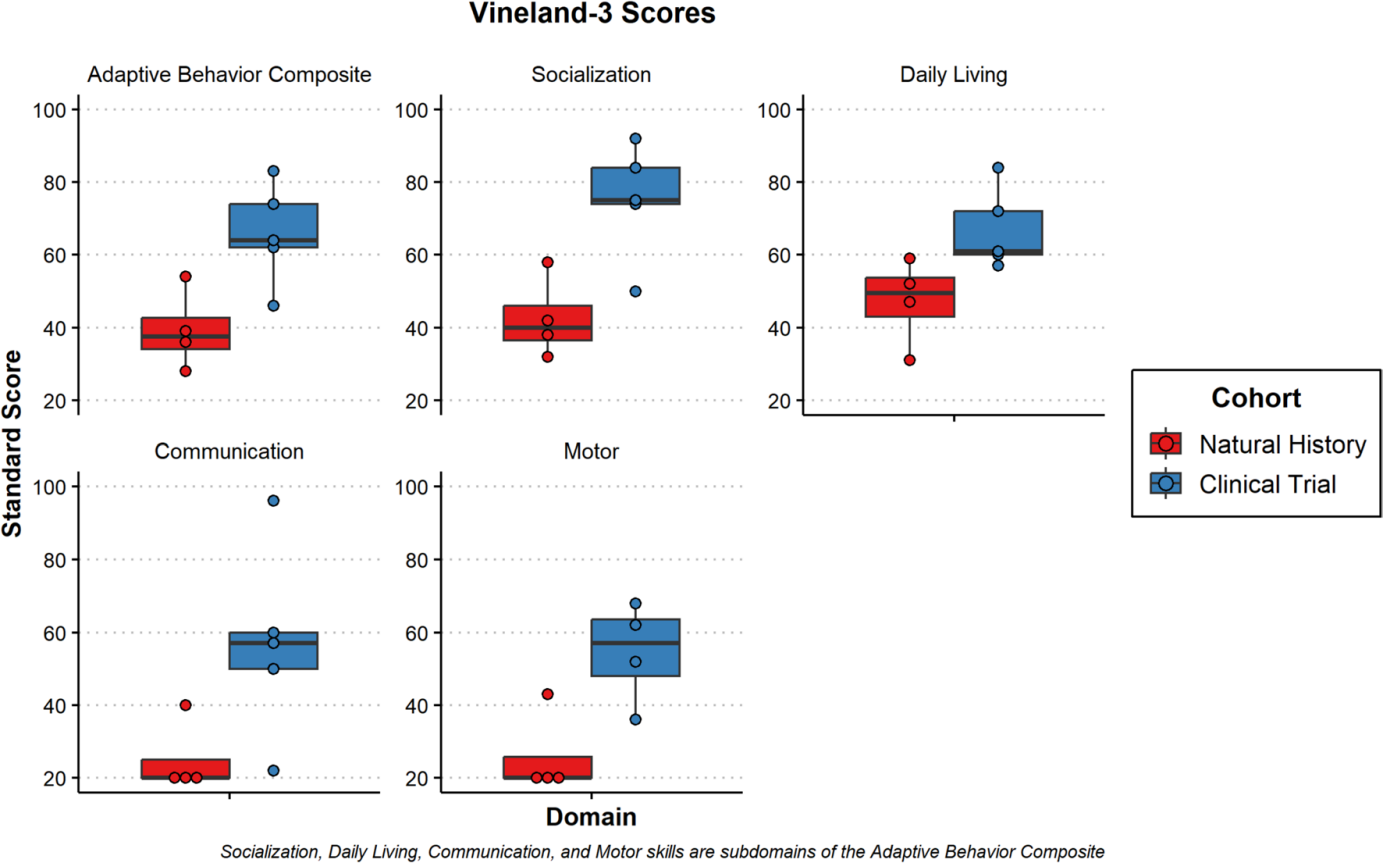
Boxplots of standard scores from the vineland-3 for subjects 1–8

**Fig. 5:**
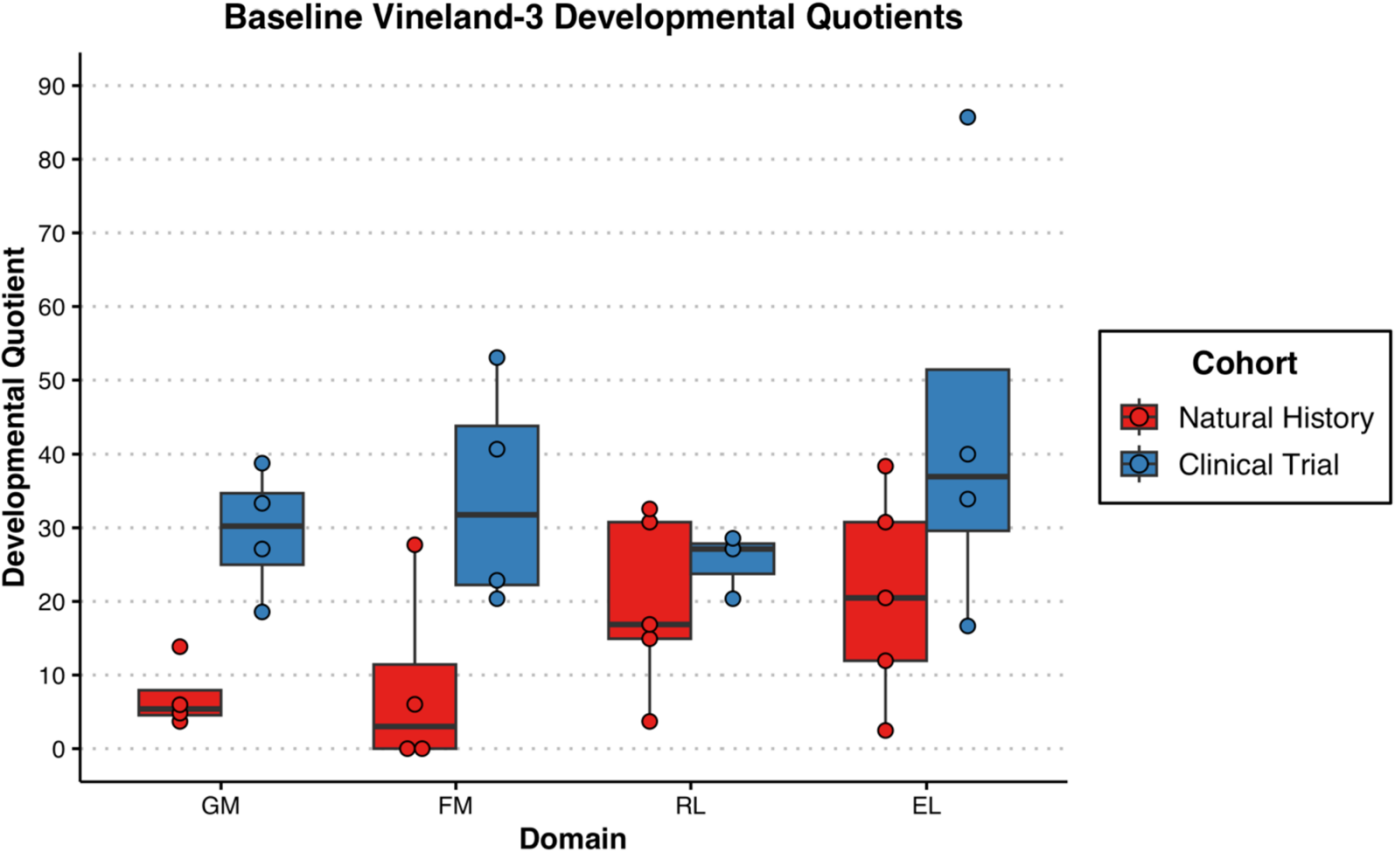
Boxplots of developmental quotients scores from the vineland-3 for subjects 1–8

With respect to adaptive functioning, there was noted to be significant variability although all but participant 8 was noted to be in the exceptionally low range on the Vineland-3 Adaptive Behavior Composite (Standard Score mean = 51.5 ± 31.5). Composite standard scores for the adaptive measure are presented in Fig. 4. Participants in the clinical trial were rated overall at a higher level of functioning than the participants in the natural history portion. Similar to results from the Mullen, follow-up evaluations on the Vineland-3 for participants 1, 2, 5 and 6 also demonstrated parent-reported regression overall (Adaptive Behavior Composite; see supplementary Fig. 2). Individually, participant 5 had sharp regressions in Communication (slope of − 31) while the others remained at the floor of the subdomain (participants 1 and 2 had slopes of 0 and participant 6 a slope of − 2). Daily Living remained stable for participants 1 (slope of 0) and 5 (slope of 1) but was noted to regress for participants 2 (slope of − 16) and 6 (slope of − 17). Social skills were also relatively stable for participants 1 (slope of 2) and 2 (slope of − 4) but showed a sharper decline in 5 and 6 (both with slopes of − 18). Further, Motor Skills were already at the floor for participants 1 and 2 (both with slopes of 0) but demonstrated rapid regression for participants 5 (slope of − 26) and 6 (slope of − 32). A look at the Developmental Quotients for the motor and communication subscales of the Vineland-3 revealed smaller differences between natural history and clinical trial participants. Further, the language scales were rated more highly than motor scales (Fig. 5).

Figure 4 Boxplots of Standard Scores from the Vineland-3 for subjects 1–8. The scores collected are the Adaptive Behavior Composite made up of the other subdomains including Socialization, Daily Living Skills, Communication, and Motor Skills composites. Subjects from the natural history study (NH) are colored red, and subjects from the gene therapy clinical trials (Clinical) are colored blue

For the clinical trial portion, assessment of health-related quality of life was assessed (participants 5–8). Three participants received the ITQOL (participants 5, 6, and 8). There was significant variability in responses (Supplementary Fig. 3), but parents rated Overall Health as good to excellent. While parents for participants 6 and 8 rated their child’s Change in Health much worse than one year ago, parents of participant 5 rated it to be about the same. Parental Emotional Impact and Family Cohesion also revealed significant variability. The QI-Disability was administered to participants 5 and 7 (Supplementary Fig. 4). The greatest variability was seen in Negative Emotions (e.g., behavioral outbursts, withdrawn behavior), but both families rated generally positive Physical Health, Positive Emotions (e.g., smiling, laughing), Social Interaction, and Leisure Skills.

Figure 5 Boxplots of Developmental Quotients Scores from the Vineland-3 for subjects 1–8. The scores represented are the Gross Motor (GM), Fine Motor (FM), Receptive Language (RL) and Expressive Language (EL) subscales. Subjects from the natural history study (NH) are colored red, and subjects from the gene therapy clinical trials (Clinical) are colored blue

## Discussion

Clinical reports and research in CLN7 have documented neurodevelopmental regression in cognitive, language and motor abilities along with seizures [12, 13]. We reviewed the current literature to note the 67 different pathogenic variants are described in 74 patients (See Supplementary Table 1) [14–38]. Our comprehensive cross-sectional and retrospective review outlines valuable information integrating the clinical findings with standardized neuropsychological assessments, electrophysiologic and neuroimaging findings. Although our sample size is small, it shows a geographically and culturally diverse cohort of patients.

In this study, median age of onset is 3.4 years with median age of diagnosis at 4.8 years. It is also important to note that onset of symptoms was noted no later than 4 years of age in all patients (8/8). Patients had a median diagnostic delay of 18 months from symptom onset to diagnosis, which suggests a delay in recognition by clinicians. This delay in diagnosis of CLN7 is due to the rarity of CLN7 deficiency, paucity of clinical data reported in current literature and lack of awareness. With this study data, we aim to add to descriptive phenotype of CLN7 disease through standardized neuropsychological and quality of life assessment tools.

Common first symptom reported by the family which led to seek medical attention was gait disturbance in 2 patients, “shaking” and falling in 2 patients, language difficulty or delay in 2 patients. it is also important to note that paucity in language development was reported in 6/8 patients. This plateau in language was noted right after the age of 2 years, however not significant enough to seek medical attention.

In the present study all patients developed seizures, followed by relentless neurologic regression with loss of motor and language functions. Electroencephalogram (EEG) findings in variant late infantile neuronal ceroid lipofuscinosis type 7 (CLN7) have been poorly described in the literature. In our case study of eight patients, video-EEG findings were non-specific and consistent with a multifocal and/or generalized epileptic encephalopathy with an elevated risk for seizures. Two epileptic seizures were captured in two different patients and were identified as a myoclonic and an atonic seizure. Ictal EEG showed a bi-occipital non-lateralizing onset triggered by photic stimulation and a generalized ictal onset, respectively. Further studies are needed to reach a more conclusive description of electro-clinical patterns of patients with CLN7.

Abnormal MRI results were observed in all (8/8) cases reviewed. All patients had generalized findings with diffuse cortical and cerebellar atrophy with white matter involvement. Diffuse white matter involvement with T2 hyperintensities were noted in 7/8 cases and periventricular gliosis was reported in 2/8 cases. Thalami were involved in all cases with a variable degree of volume loss and gliosis. Cognitively and adaptively, participants had declines in functioning seen both when compared to peers as well as longitudinally for those with follow-up data. Participants in the natural history portion also performed more poorly than those in the clinical trial thought to be secondary to longer effects of the disease.

The date of both natural history and Ct patients is presented here to strengthen the numbers for the study. In rare diseases like CLN7, progression of the disease is poorly reported with paucity in natural history knowledge in current literature, specifically data pertaining to survival rates per year after the onset of the disease. On one hand the data from clinical trial cohort strengthens the disease description as we see similar pattern of disease burden. On the other hand, however, in clinical trial cohort, the patients had relatively higher level of functioning at the start of the study due the eligibility criteria that they had to meet.

In our study, the youngest patient in natural history study was 5 years 5 months and oldest 6 years 11 months. The age range for patients in clinical trial cohort was 4 years 1 month to 5 years 11 months. The age of onset ranged from 2 years and 4 months to 4 years while in clinical trial cohort, the age of onset of symptoms ranged from 2 to 3 years 8 months. Similarly, there were no major differences noted in age of onset of seizures between the two cohorts. As noted above, there is mild age gap which could have contributed to the difference noted between the two cohorts, but we are unable to do head-to-head comparison due to lack of prospective data for natural history cohort. Moreover, it will be of huge interest to review the clinical data specifically survival data for all the patients.

Various factors that could potentially affect the disease progression and severity are age of onset of first symptoms along with age of onset of seizure, age of onset of developmental regression and velocity of developmental regression, however further studies are needed specifically a prospective natural history study to elaborate the importance of these datapoints. This will not only help with clinical care and management of these patients but also serve as a control arm for clinical trial.

The study limitations include retrospective nature and small sample size and missing data. Another limitation is lack of long term follow up of these patients. Further, due to decline in patients, there are very limited tools to assess their neurodevelopmental abilities. While the use of raw scores and developmental quotients helped to be able to better capture abilities, there are no current tests that can fully capture the lower end of the disease spectrum. There are also linguistic and cultural considerations for such a rare disease that are difficult to overcome in a singular study. Our considerations for this are further detailed in Bordes Edgar et al. [11]

## Conclusions

This study provides a comprehensive description of CLN7 disease, using medical history questionnaires and standardized tools. There is description of more than 121 patients in literature (Supplemenatry Table 1) based upon retrospective chart reviews. Our study adds data to elaborate on clinical phenotype, neuroimaging and electrophysiologic findings along with standardized neuropsychological assessments. We aim to explore the role of standardized testing that can not only describe the depth and breadth of disease phenotype but can also be used as clinical endpoint points in a future clinical trial. We believe the data from this study would be instrumental with future therapy trials as it provides a baseline for assessment of improvement. A prospective natural history study would provide even more useful information for clinical and further research and would be the next step for future studies.

## Supplementary Information

Additional file 1

## Data Availability

The data were extracted from the participants electronic medical records as well as paper source data from participation in the natural history study and clinical trial. The datasets generated during and/or analyzed during the current study are not publicly available due to protection of participants privacy and protected health information. A password protected; de-identified data set stored on the secure servers at Childrens Health can be available from the corresponding author on reasonable request.
