# Supplementary material for: Neuronal ceroid lipofuscinoses type 7 (CLN7): a case series reporting cross sectional and retrospective clinical data to evaluate validity of standardized tools to assess disease progression, quality of life, and adaptive skills
Source: Orphanet J Rare Dis. 2024 Dec 19;19:468. doi: 10.1186/s13023-024-03448-8 (PMC11657365; doi:10.1186/s13023-024-03448-8)
Supplement: Supplementary file 1 — Additional file 1 [file 13023_2024_3448_MOESM1_ESM.docx]

**Supplementary Materials:**

Supplementary Figure 1: Dot plot of the Mullen Standard Scores and Developmental Quotients for 2 subjects


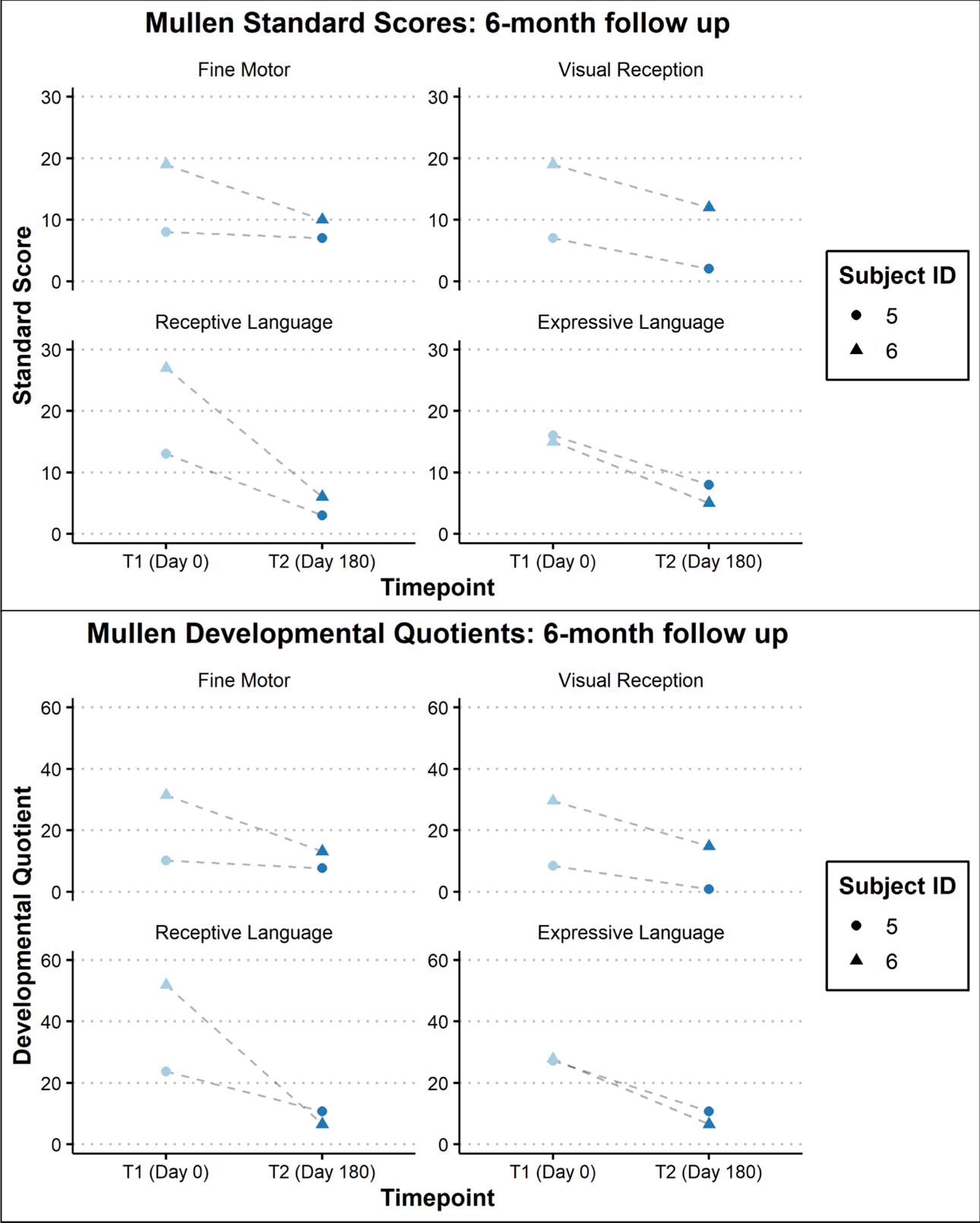


*Supplementary Figure 1 Dot plot of the Mullen Standard Scores and Developmental Quotients for 2 clinical trial subjects from the baseline data (T1) and after the course of 1 year (T2).*

Supplementary Figure 2: Dot plot of the Vineland-3 composite and domain scores baseline to 1 year follow up:


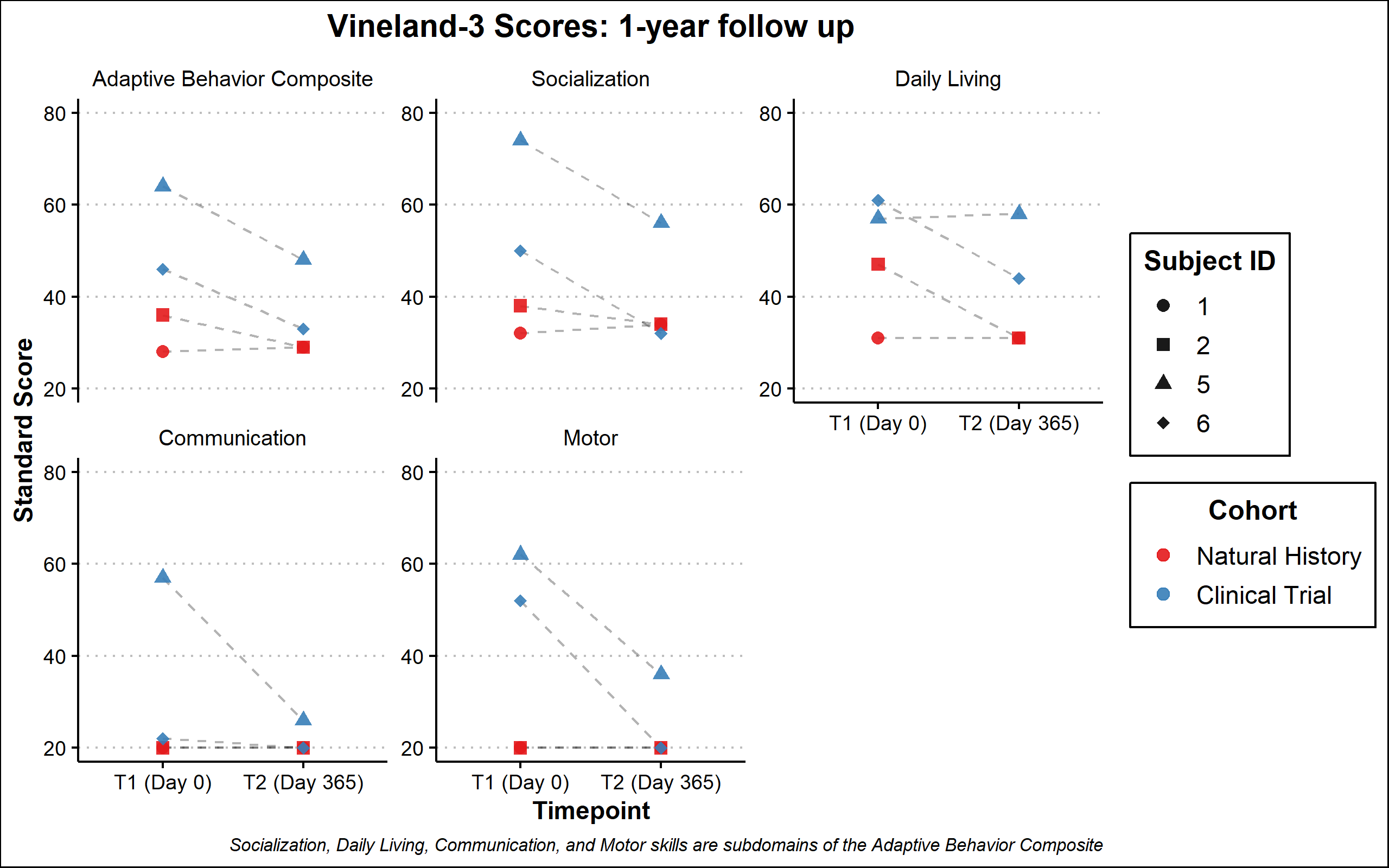
*Supplementary Figure 2 Dot plot of the Vineland-3 composite and domain scores for 2 natural history subjects (ID: 1 and 2) and 2 clinical trial subjects (ID: 5 and 6) from the baseline data (T1) and after the course of 1 year (T2). Scores represented are the Adaptive Behavior Composite made up of the other subdomains including Socialization, Daily Living Skills, Communication, and Motor Skills composites.*

Supplementary Figure 3: Boxplots of the ITQOL scores for 3 clinical trial subjects.


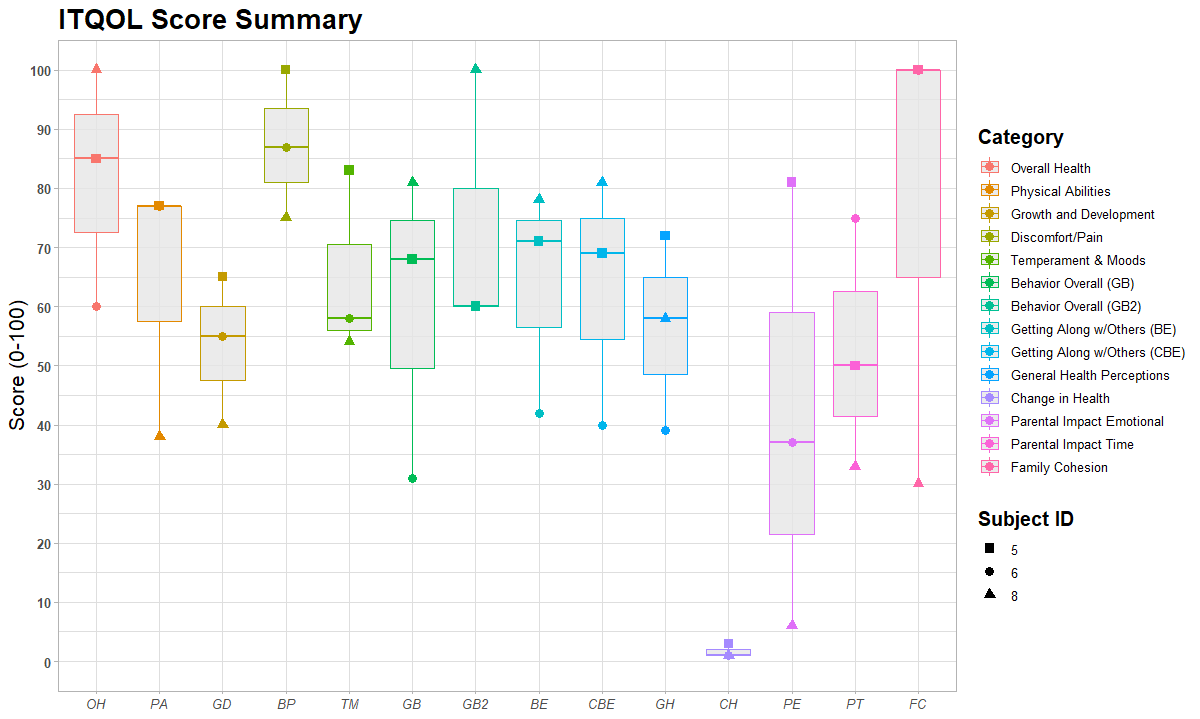


*Supplementary Figure 3 Boxplots of the ITQOL scores for 3 clinical trial subjects. Scores represented are Overall Health (OH), Physical Abilities (PA), Growth and Development (GD), Discomfort/Pain (BP), Temperament and Moods (TM), Behavior Overall (GB & GB2), Getting Along with Others (BE & CBE), General Health Perceptions (GH), Change in Health (CH), Parental Impact Emotional (PE), Parental Impact Time (PT), and Family Cohesion (FC).*

Supplementary Figure 4: Boxplots of the QI-Disability scores for 2 clinical trial subjects


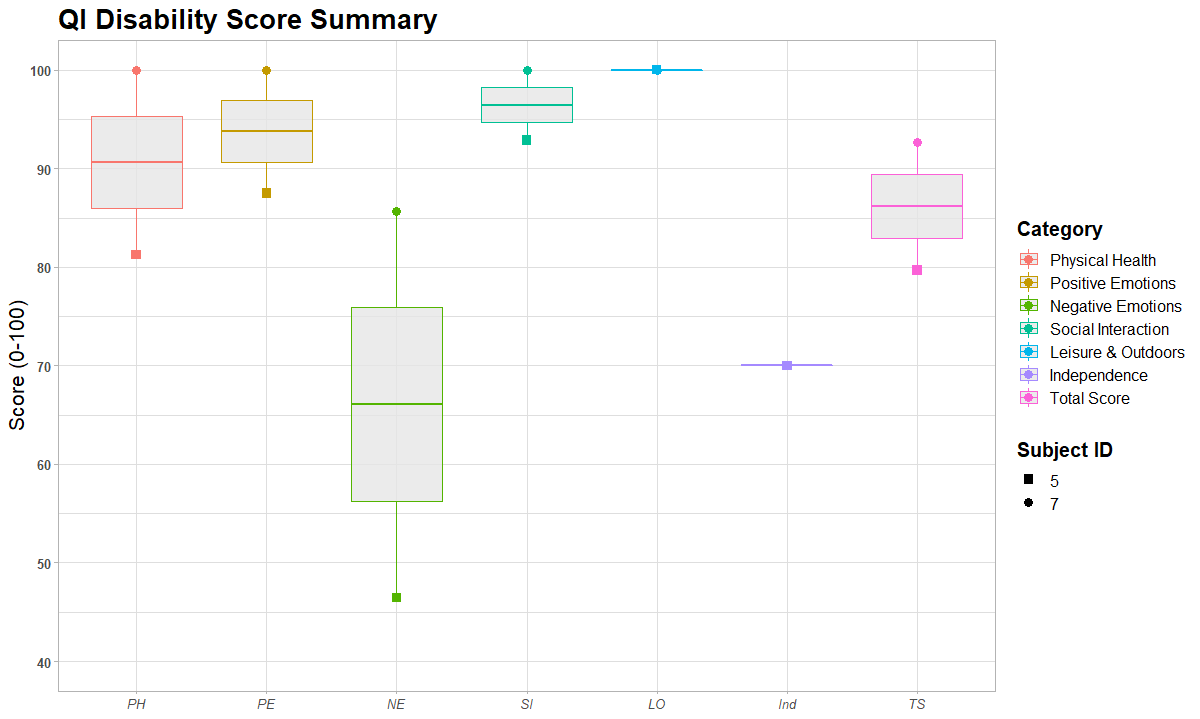


*Supplementary Figure 4: Boxplots of the QI-Disability scores for 2 clinical trial subjects. Scores represented are Physical Health (PH), Positive Emotions (PE), Negative Emotions (NE), Social Interaction (SI), Leisure & Outdoors (LO), Independence (Ind), and Total Score (TS).*

Supplementary Table 1: *MFSD8* Gene Mutations in the Literature

| Mutation, nucleotide change^11^ | Mutation, protein change | Number of Patients with Mutation | Patient Location | Citations | Citation Number in List |
| --- | --- | --- | --- | --- | --- |
| c.863+3_4insT | Altered Splicing | 3 | Italy | Aiello *et al.,* 2009 | 5 |
| c.929G>A | p.Gly310Asp | 4 | Turkey; Italy; France | Topçu *et al.*, 2004; Siintola *et al.*, 2007; Aiello *et al.*, 2009; Poncet *et al.,* 2022 | 14; 1; 5; 15 |
| c.627_643del17 | p.Met209IlefsX3 | 1 | Italy | Aiello *et al.*, 2009 | 5 |
| c.154G>A | p.Gly52Arg | 1 | Italy | Aiello *et al.*, 2009 | 5 |
| c.1444C>T | p.Arg482* | 4 | France; China; Scandinavian; United States | Aiello *et al.*, 2009; Ren, 2019; This Study | 5; 16 |
| c.1141G>T | p.Glu381* | 7 | France; Netherlands | Aiello *et al.,* 2009; Roosing *et al.,* 2015; Poncet *et al.,* 2022 | 5; 4; 15 |
| c.863+1G>C | Altered Splicing | 2 | Italy; Turkey | Aiello *et al.*, 2009; Kousi *et al.*, 2009 | 5; 2 |
| c.2T>C | p.Met1Thr | 1 | Italy | Aiello *et al.*, 2009 | 5 |
| c.1340C>T | p.Pro447Leu | 1 | Italy | Aiello *et al.*, 2009 | 5 |
| c.881C>A | p.Thr294Lys | 27 | Italy; Turkey; Czech Republic; Romania; Egypt; Hungary; France | Aiello et al., 2009; Kousi et al., 2009; Craiu et al., 2015  Refeat et al., 2022; Poncet *et al*., 2022; Jilani, *et al.,* 2019; This Study | 5; 2; 17; 18; 15; 19 |
| c.103C>T | p.Arg35* | 6 | Italy; Turkey; Argentina; England; Paraguay | Aiello *et al.*, 2009; Kousi *et al.*, 2009; Kohan *et al.*, 2015; Khan *et al.*, 2017; This Study | 5; 2; 20; 21 |
| c.1398C>T | p.Pro412Leu | 3 | Saudi Arabia | Aldahmesh *et al.*, 2009 | 12 |
| c.416G>A | p.Arg139His | 4 | India; Egypt | Kousi *et al.*, 2009; Refeat *et al*., 2022; Jilani, *et al.,* 2019 | 2; 18; 19 |
| c.468_469delinsCC | p.Thr156_Ala157delins, Thr156_Pro157 | 1 | Netherlands | Kousi *et al.*, 2009 | 2 |
| c.627_643del | p.Met209Ilefs*3 | 2 | Italy | Aiello *et al.*, 2009; Kousi *et al.*, 2009 | 5; 2 |
| c.1103-2delA | Altered Splicing | 1 | Czech Republic | Kousi *et al.*, 2009 | 2 |
| c.1393C>T | p.Arg465Trp | 1 | Albania/Greece | Kousi *et al.*, 2009 | 2 |
| c.259C>T | p.Gln87* | 1 | Canada | Kousi *et al.*, 2012 | 3 |
| c.479C>A | p.Thr160Asn | 1 | Turkey | Kousi *et al.*, 2012 | 3 |
| c.479C>T | p.Thr160Ile | 2 | Cook Islands; Egypt | Kousi *et al.*, 2012; Refeat *et al.,* 2022 | 3; 18 |
| c.554-1G>C | Altered Splicing | 1 | Romania | Kousi *et al.*, 2012 | 3 |
| c.754+1G>A | Altered Splicing | 2 | Turkey | Kousi *et al.*, 2012 | 3 |
| c.1373C>A | p.Thr458Lys | 2 | Romania | Kousi *et al.*, 2012; This Study | 3 |
| c.1394G>A | p.Arg465Gln | 5 | Turkey; England | Kousi *et al.*, 2012; Khan *et al.,* 2017; Jilani, *et al.,* 2019 | 3; 21; 19 |
| c.1408A>G | p.Met470Val | 1 | Turkey | Kousi *et al.*, 2012 | 3 |
| c.1420C>T | p.Gln474* | 1 | Turkey | Kousi *et al.*, 2012 | 3 |
| c.472G>A | p.Gly158Ser | 5 | Israel | Mandel *et al.*, 2014 | 22 |
| whole gene deletion | whole gene deletion | 1 | China | Qiao *et al.*, 2022 | 23 |
| c.136_137delAT | p.Met46fs | 1 | China | Qiao *et al.*, 2022 | 23 |
| c.750A>G | Altered Splicing | 4 | Turkey; France | Reith *et al.*, 2022; Poncet *et al*., 2022 | 24; 15 |
| c.697A>G | p.Arg233Gly | 1 | Turkey | Topçu *et al.*, 2004; Siintola *et al.*, 2007 | 14; 1 |
| c.754+2T>A | Altered Splicing | 14 | Turkey; Czech Republic; Romania; Hungary | Topçu *et al.*, 2004; Siintola *et al.*, 2007; Kousi *et al.*, 2009; Craiu *et al.*, 2015; Jilani, *et al.,* 2019; This Study | 14; 1; 2; 17; 19 |
| c.894T>G | p.Tyr298* | 1 | India | Siintola *et al.*, 2007 | 1 |
| c.1102G>C | p.Asp368His | 3 | Turkey; Netherlands; United States | Siintola *et al.*, 2007; Roosing *et al.*, 2015; Kim *et al.*, 2019 | 1; 4; 25 |
| SVA insertion | Altered Splicing | 1 | United States | Kim *et al.*, 2019 | 25 |
| c.1286G>A | p.Gly429Asp | 1 | Turkey | Topçu *et al.*, 2004; Siintola *et al.*, 2007 | 14; 1 |
| c.362A>G | p.Tyr121Cys | 3 | Egypt | Stogmann *et al.*, 2009 | 13 |
| c.1361T>C | p.Met454Thr | 14 | India; Turkey; Iran | Patiño *et al.*, 2014; Khan *et al.*, 2017; Zare-Abdollahi *et al.*, 2019 | 26; 21; 32 |
| c.1219T>C | p.Trp407Arg | 3 | India | Patiño *et al.*, 2014 | 26 |
| c.1006G>C | p.Glu336Gln | 15 | Netherlands; England; France | Roosing *et al.*, 2015; Khan *et al.,* 2017; Poncet *et al.,* 2022 | 4; 21; 15 |
| c.233G>A | p.Trp78* | 2 | England | Khan *et al.*, 2017 | 21 |
| c.554-5A>G | Altered Splicing | 1 | China | Ren *et al.*, 2019 | 16 |
| c.525T>A | p.Cys175* | 1 | Russia | Kozina *et al.*, 2018 | 27 |
| c.325_339del | p.Val109_Ile113del | 1 | Iran | Bereshneh & Garshasbi, 2018 | 28 |
| c.439+3A>C | p.Ile67Glufs*3 | 1 | not reported | Bauwens *et al.*, 2019 | 29 |
| c.590del | p.Gly197Valfs*2 | 1 | not reported | Bauwens *et al.*, 2019 | 29 |
| c.721G>T | p.Gly241* | 1 | Turkey | Kose *et al.*, 2021 | 30 |
| c.1445G>C | p.Arg482Pro | 1 | Germany | Birtel *et al.*, 2018 | 31 |
| c.1235C>T | p.Pro412Leu | 8 | Iran; Egypt | Zare-Abdollahi *et al.*, 2019 ; Refeat *et al.,*  2022 | 32; 18 |
| c.1093C>T | p.Gln365* | 2 | Turkey | Kose *et al.*, 2021 | 30 |
| c.1391C>T | p.Ala464Val | 1 | Poland | Ziora-Jakutowicz *et al.*, 2019 | 33 |
| c.301G>C | p.Ala146Pro | 1 | Turkey | Kose *et al.*, 2021 | 30 |
| c.63-1G>A | Altered Splicing | 2 | India; not reported | Gowda *et al.,* 2020; Dozières-Puyravel et al., 2020 | 34; 35 |
| c.886G>C | p.Asp269His | 1 | Egypt | Refeat *et al.,* 2022 | 18 |
| c.600G>A | p.Trp200Ser | 1 | Egypt | Refeat *et al.,* 2022 | 18 |
| c.863 + 2dup (G>A) | Altered Splicing | 1 | Italy | Pasquetti *et al.,* 2023 | 36 |
| c.850G>C | p.Ala284Pro | 1 | Bangladesh | Rahman *et al.,* 2021 | 37 |
| c.1241_1242insGAAT | p.Ile414Metfs*109 | 1 | Not reported | Jilani *et al.,* 2019 | 19 |
| c.863+4A>G | not reported | 1 | Not reported | Jilani *et al.,* 2019 | 19 |
| c.1351-G>A | p.Phe186Cys | 1 | China | Niu *et al.,* 2022 | 38 |
| c.557T>G | p.Phe186Cys | 1 | China | Niu *et al.,* 2022 | 38 |
| c.755-2726_998+1981delinsGTA | p. Ser253Leufs*79 | 1 | France | Poncet *et al.,* 2022 | 15 |
| c.104G>A | p.Arg35Gln | 1 | France | Poncet *et al.,* 2022 | 15 |
| c.155G>C | p.Gly52Ala | 1 | France | Poncet *et al.,* 2022 | 15 |
| c.1265C>A | p.Ser422* | 1 | France | Poncet *et al.,* 2022 | 15 |
| c.1009C>T | p.Arg337Cys | 1 | France | Poncet *et al.,* 2022 | 15 |
| c.998+1669A>G | p.Lys333Asnfs*18 | 1 | France | Poncet *et al.,* 2022 | 15 |
